# The Power of Packaging: A Scoping Review and Assessment of Child-Targeted Food Packaging

**DOI:** 10.3390/nu12040958

**Published:** 2020-03-30

**Authors:** Charlene Elliott, Emily Truman

**Affiliations:** Department of Communication, Media, and Film, University of Calgary, Calgary, AB T2N 1N4, Canada; emily.truman@ucalgary.ca

**Keywords:** children, nutrition, food packaging, food marketing, power, exposure, marketing techniques, youth, childhood obesity

## Abstract

Child-targeted food marketing is a significant public health concern, prompting calls for its regulation. Product packaging is a powerful form of food marketing aimed at children, yet no published studies examine the range of literature on the topic or the “power” of its marketing techniques. This study attempts such a task. Providing a systematic scoping review of the literature on child-targeted food packaging, we assesses the nutritional profile of these foods, the types of foods examined, and the creative strategies used to attract children. Fifty-seven full text articles were reviewed. Results identify high level trends in methodological approaches (content analysis, 38%), outcomes measured (exposure, 44%) and with respect to age. Studies examining the nutritional profile of child-targeted packaged foods use various models, classifying from anywhere from 41% to 97% of products as unhealthy. Content analyses track the prevalence of child-targeted techniques (cartoon characters as the most frequently measured), while other studies assess their effectiveness. Overall, this scoping review offers important insights into the differences between techniques tracked and those measured for effectiveness in existing literature, and identifies gaps for future research around the question of persuasive power—particularly when it comes to children’s age and the specific types of techniques examined.

## 1. Introduction

In February 2020, a team of global experts released “A Future for the World’s Children”—a report jointly produced by the World Health Organization, UNICEF and The Lancet that outlined “urgent and actionable agendas” to support child health and well-being [1] (p.4). Among other things, the report draws attention to the “severe threats” posed to children by “harmful commercial marketing”, such as the marketing of unhealthy food to children [1] (p. 26), and calls for its regulation.

“A Future for the World’s Children” joins many other reports, initiatives and policies that affirm the need to protect children from the marketing of foods high in sugar, fat and/or salt [2,3]. Concern over the negative effects of food marketing on children’s health has been steadily climbing since the first systematic review on the topic was published in 2003 [4]. Currently, 16 countries have statutory regulations on unhealthy food marketing to children [3]. Yet the call to regulate is complex because *food marketing* captures multiple communication platforms (from television advertising and digital media to the physical environment and product packaging) and multiple marketing strategies or techniques (from spokes-characters, premium offers and health/nutrition related claims to emotional appeals and themes of fun or taste). While similar marketing techniques may be found across different “media”, those media are most certainly not the same, and some communications platforms are far more studied, understood, and evoked than others. Television advertising, for example, has dominated the studies on food marketing to children [4], and (according to a recently published scoping review) it remains the most frequently analysed platform in the recent literature [5]. Even the “Future for the World’s Children” report, in calling to regulate food marketing to children, identifies a narrow subset of marketing; namely, television advertising and that on social media. Simply put, certain forms of food marketing to children are (comparatively) overlooked. Product packaging, in particular, warrants closer attention. It may be less studied than televised food advertising to children, but this does not make it less powerful.

Unlike television or digital advertising, packaging is essential to decision making at the point of sale. Child-targeted food packaging is itself an advertisement, requiring its own, unique considerations. As such, the purpose of this study is to enhance understanding of the overall “power” of child-targeted food packaging. To this end, we summarize the literature examining child-targeted food packaging to assess, in particular, the nutritional profile of these foods, the types of foods examined, and the creative strategies used to attract children.

Guiding this research is a consideration of the World Health Organization’s (WHO) Set of Recommendations on the Marketing of Foods and Non-Alcoholic Beverages to Children [2] (see also [6]), which suggests that effective marketing communications is a function of exposure (the frequency and reach of a message) and power (the content, design and creative strategies used to target and persuade) [6] (p.10). While a strong body of evidence documents how food marketing exposure impacts children [7], “comparatively few studies examine the creative content of food advertising which plays a critical role in its persuasive effect” [7] (p. 560). Only very recently has literature sought to capture the treatment of power when it comes to food marketing to children more broadly [5]. To date, however, no studies have specifically focused on what the corpus of literature on food packaging directed at children reveals about its nutritional quality, types of foods examined, impact, and persuasive appeals. Our study addresses this research gap, providing a scoping review of literature examining child-targeted food packaging and assessing it with respect to persuasive power.

## 2. Methods

A systematic literature search was conducted in eight databases (Scopus, Web of Science, Embase, MEDLine, CAB Abstracts, CINHAL, PubMED, JSTOR) for all available dates (up to December 31, 2019), to locate peer-reviewed literature on child-targeted food packaging. The following search string was used: food packaging AND child* AND (food marketing OR food promotion). No restrictions were placed on publication type or language. Inclusion criteria required that child-targeted food packaging was the main focus of the study, and one or more food marketing techniques/strategies on those packages was examined. That is, the study needed to focus on child-targeted food packaging *and* its marketing techniques or “power”. Other inclusion criteria were that the populations (if examined as part of the study) were children between 2–12 years. This age range was selected as it is used by several countries with statutory regulations on unhealthy food marketing to children, and is also the proposed range for regulations in Canada. (Note that ages slightly below and above this range are documented in the captured data. Parents may also be part of the study.)

A hand search was then performed based on the references cited in the included studies. Additionally, we conducted a Google Scholar search in December 2019 to obtain relevant grey literature (i.e., white papers and organizational reports). As per the recommended protocol for grey literature searches on Google Scholar, we reviewed the first 800 abstracts returned [8].

**Figure 1:**
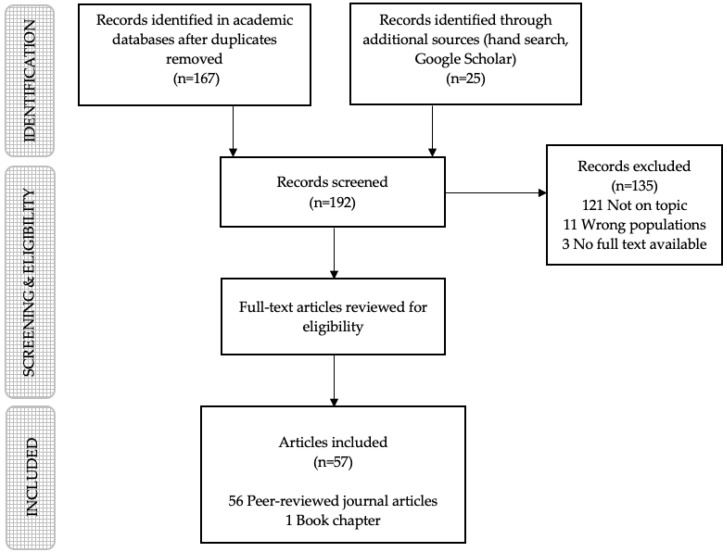
Literature search flow chart.

One hundred and sixty seven records were located in the initial search, and 135 were excluded for the following reasons: not on topic (i.e., child-targeted food packaging and the power of its marketing techniques is not a central focus of the study), *n* = 121; examines wrong population (i.e., parents or teenagers), *n* = 11; and no full text available, *n* = 3. The hand search located seven additional abstracts, while the Google Scholar search identified 18 additional peer-reviewed journal articles, but no relevant grey literature. See Figure 1 for a flow chart of the search process.

Included studies had data extracted for: study type, methods, object of study (i.e., physical food packaging, images of packaging, etc.), population age range, outcomes measured, nutrient profiling models used, key findings on nutritional quality of child-targeted foods, food types tracked (i.e., cookies, crackers, yogurts, etc.), indicators used and their sources (i.e., criteria used by researchers to identify and analyze child-targeted packaging), key findings on the “power” of child-targeted marketing techniques on packaging (i.e., types, prevalence, influence or impact), and definitions of “power”. The type of journal publishing each article (i.e., field of study) was also documented.

## 3. Results

Fifty-seven studies are included in this scoping review [9,10,11,12,13,14,15,16,17,18,19,20,21,22,23,24,25,26,27,28,29,30,31,32,33,34,35,36,37,38,39,40,41,42,43,44,45,46,47,48,49,50,51,52,53,54,55,56,57,58,59,60,61,62,63,64,65]. The first study on children and packaged foods was published in 1978 in the Journal of Marketing: it explored parent-child interactions in the supermarket (specifically related to cereal requests) [12], with an eye to recommending how to best direct advertising and promotion efforts for child-oriented cereals. It took 25 years for the next published study on children and packaged foods to materialize—also a marketing study on cereals with an eye to discussing “implications for brand/packaging management” [50] (p. 419). Only in 2006 does the public health literature first address child-directed food packaging with a study on the extent and nature of food promotion directed to children in Australian supermarkets [18]—a study which is assessed by its authors not for its ability to provide marketing strategies, but rather in light of the nutritional quality of the food and the need to consider appropriate guidelines when it comes to targeting children. Interest in child-targeted food packaging spikes starting in 2012, with 68% of the literature assessed appearing between 2012 and 2019, at an average of five studies published per year.

While the earliest study in our dataset was published in a marketing journal, the majority of the studies come from the field of health/public health (63%), followed by marketing/business literature (19%), and critical consumer theory/communication (18%). Fifty eight percent of the included studies are cross-sectional; the rest are descriptive. The majority of studies (74%) use physical food packaging (compared to printed photographs of packaging or digital images) as their object of study, and content analysis (38%) is the most prevalent methodological approach. When it comes to age studied, no consistency is found in terms of age range used across the studies. For example, some studies include participants ranging from age 1–12 [19], 0–13 [52], and 0–16 [53], respectively, while others focus solely on age four [55], age six [65], or span five years [11,42,49]. The most frequently studied ages in the child populations identified fall between two and 12 years (42%). Finally, over all of the included studies, exposure is the most frequently measured outcome (44%). See Table 1 for a summary of methodological approaches, objects of study, population age ranges, and outcomes measured across all included literature.

### 3.1. Nutritional Quality of Child-Targeted Packaged Foods and Types of Foods Examined 

The nutritional quality of child-targeted packaged foods is evaluated in the content analyses (*n* = 22) identified. Twenty-one of the content analyses used various models (some more than one) to assess the healthfulness of child-targeted packaged foods, including at least 13 different sets of criteria. Of the nutrient models used, the UK’s Nutrient Profiling Model is the most prevalent (used by five studies). Table 2 outlines the nutrient profiling criteria used in each of the content analysis, listed chronologically in order to reveal the evolution of nutrition criteria over time. Since the finalized Pan American Health Organization (PAHO) nutrient profiling model was only published in 2016, for example, it makes sense that it does not appear in a content analysis until 2017.

Despite the range of criteria used, all of the content analyses document the poor nutritional quality of child-targeted foods, with studies reporting up to 97% of the products analysed as unhealthy [17]. Even the analyses that specifically isolate the “better-for-you” food products designed to appeal to children revealed a poor nutritional profile, with 65% of “better-for-you” products classified as high-fat, salt or sugar [26]. However, certain caveats prove necessary. Sample sizes in the content analyses vary substantially, ranging from 69 products up to 1005, and the studies examine different categories of food (from cereal, to candy, to beverages, to pastries and cookies), making comparison across studies inappropriate. Some studies analyse all child-directed foods found in the supermarket [9], while others analyse the “regular” foods that have been repackaged to appeal to children, specifically excluding “junk food” (such as confectionary/candy/chocolate, sugary sodas and salty snacks) [22,23,26,30]; and some isolate specific categories or types of food—such as cereals [50,52,54,57,63], beverages [51] or snack food, confectionary and “dairy snacks”/ice cream [18] (See Table 2).

The most prevalent food types documented in the content analyses evaluating the nutritional quality of child-targeted foods were in the dry goods section, including cereals and chocolate/confectionary products (Table 2). Yet, to echo the previous caveat, one cannot simply compare findings across the content analysis studies since some of the samples were entirely comprised of cereals, or focused on a limited range of products.

Beyond the content analyses, the experimental, mixed methods, focus group, survey, interview-based, and observational studies also used a wide variety of food types in analysing the impact of child-directed packaging. Experimental studies (*n* = 20), for example, focused on specific packaged food products such as yogurt and sponge cake [10,11], chocolate wafers [65], soy snack food bars [42], orange juice [14], or various branded/brand name products [32]. Only two of the experimental studies (headed up by the same research team) used the exact same products—in this case, showing children printed labels for yogurt and sponge cake [10,11]. Survey studies (*n* = 2), moreover, tested the influence of packaging on either children’s perception of milk [29] or products with fruit, minimal fruit, or no fruit [48]. In short, different combinations of foods were used in virtually all of the studies analysed. However, the different types of foods used in the experimental, mixed methods, focus group, survey, interview-based and observational studies—in most cases—prove significantly less important to the outcomes found in these studies. Why? Because instead of measuring the nutritional quality of the selected packaged products, they generally sought to measure how elements on the packaging impacted attitudes (such as food preference, taste preference, perceptions of healthfulness) or behaviors (such as food choice).

### 3.2. Nutrition Claims

Not surprisingly, the majority (77%) of content analyses of child-directed food packaging tracked the prevalence of nutrition claims, although only one study [9] provided a definition. Several studies instead gave examples of such claims, such as a low-fat, source of Vitamin D, no artificial sweeteners (or other ingredient claims), or examples of health symbols [19,22,30,36,60]. In the packaged foods analysed, the frequency of nutrition/health claims ranged from 19% to 100%. Several studies assessed the nutritional quality of the products with nutrition claims, with mixed results. Four studies found that packages with nutrition claims are “healthier” products [9,20,51,53], while three studies reported that nutrition claims appear more frequently on unhealthy products [17,19,26].

### 3.3. Marketing Techniques: The “Power” of Child-Targeted Food Packaging

While the WHO defines the effectiveness of food marketing messages as a function of exposure and power [6], only two of the included studies (both content analyses) cite and briefly discuss the conception of persuasive power from the WHO [30,53]. In those studies, Elliott [30] provides specific examples of child-targeted strategies on food packaging that contribute to its power, such as fun appeals and cartoon characters, while Mehta and colleagues [53] calculate the aggregate power of those strategies, finding six or more techniques per product in their sample.

**Figure 2:**
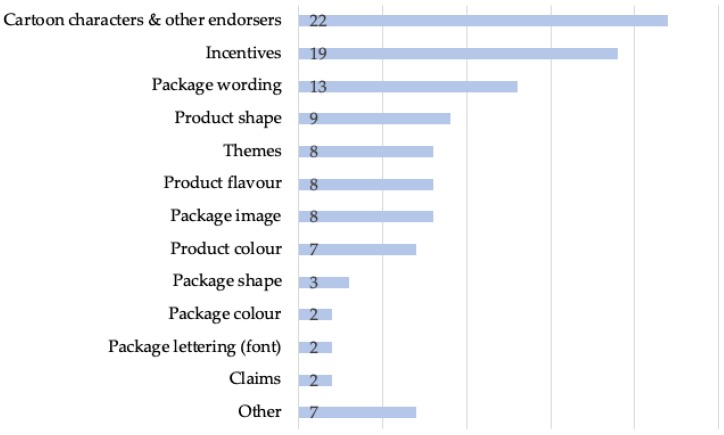
Frequency of child-targeted food packaging indicators tracked by content analyses (*n* = 22).

Even though much of the literature on child-targeted food packaging did not explicitly use the term “power”, all of the content analyses evaluate persuasive power by tracking specific marketing techniques. Presence of these techniques is examined in two ways: 1) as inclusion criteria; namely, pre-determined “indicators” used to select child-targeted food packages for analysis (*n* = 10), and 2) as analysis criteria; that is, the packaged foods are selected based on other factors (e.g., brand, food type) and analysed for the techniques applied to those packages (*n* = 12). Figure 2 presents a summary of the frequency of child-targeted indicators tracked by the content analyses (*n* = 22) in this scoping review to both identify and analyze child-specific marketing techniques on food packaging. 

Cartoon characters and other endorsers (which includes brand mascots, licensed cartoon characters, celebrities, sports figures and non-cartoon characters from TV or movies) comprise the most prevalent type of indicator measured, tracked by 100% of content analyses. An average of five indicators is measured per study (range is 1–10). Of the 10 studies that use pre-selected indicators as inclusion criteria for the products studied, the majority (*n* = 6) require that only one criteria be met for inclusion, while four of the studies require package to contain at least two criteria to qualify as “child-targeted” [22,23,53,60]. Only 41% of the content analyses cite previous studies as the source for their use of indicators, referring to a variety of peer-reviewed and grey literature on food packaging and food marketing.

**Figure 3:**
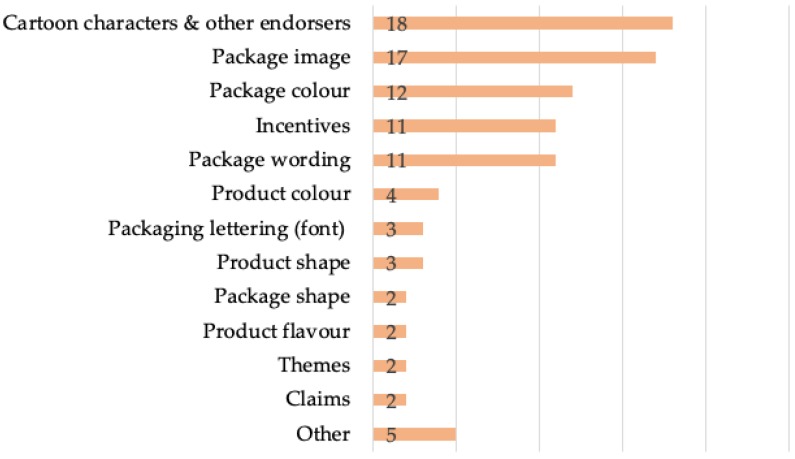
Frequency of child-targeted food packaging indicators examined in studies other than content analyses (*n* = 35).

The remaining studies captured in this scoping review examine child-targeted marketing techniques on food packaging as their stimuli for study subjects. The average number of techniques used per study varies by methodological approach, ranging from one (observational study) to six (critical research paper). The most frequently examined technique varies across study methods. Cartoon characters and other endorsers are the most prevalent technique used in experimental studies, for example, while package imagery is the most prevalent for focus groups, and incentives, package wording and imagery are the most popular in mixed methods. Figure 3 presents the variety of techniques examined across studies other than content analyses (*n* = 35).

### 3.4. Food Packaging Techniques: Prevalence and Effectiveness Assessed Across Studies

In terms of measuring persuasive power, studies in this review examined the prevalence or effectiveness of specific food packaging techniques. Across content analyses, the most prevalent technique identified in 12 of 21 studies was cartoon characters, including cartoon imagery, brand mascots and licensed characters. Variations in labelling across content analyses (from “cartoon images” to “third-party licensed character”), and the differences in sample size percentage (from 17% to 92.5%) captured in Table 3. Across all other study approaches (i.e., experimental, survey, etc.), cartoon characters were found by nine studies (39%) to be the most effective techniques impacting children’s food behaviours and attitudes, followed closely by eight studies (35%) examining the positive effects of packaging imagery and colour, and another six (26%) studies exploring the positive effects of branding identity and design. With respect to the effects measured around cartoon characters, specific outcomes varied greatly, including food intake, food choice, food preference, and taste preference, as did age ranges examined (see Table 3). 

As Table 3 reveals, packaging imagery (such as images of the products, images of ingredients and/or other visual background elements like a design or pattern) as well as colour were found to influence children’s food perceptions and preferences. For instance, children identified packages with pictures of fruit as healthier than those without [16,31,42,46], and viewed “serious looking” packaging [24], “plain packaging” [29], or packaging with “simple and clean visual elements” [42] as an indicator of healthy food. Notably, the opposite also held true. Food in “fun” packaging was perceived as less healthy by children [31]—even when the foods were plain, low fat milk [29] or sliced apples [59]. When it comes to packaging, moreover, “colour” [45] and “bright colours” [59] are discussed as influencing children’s preference. Colourful wrappings can even influence the taste preferences of preschoolers [32] and the food perceptions of older children. Specifically, children were found to associate bright colours on packaging with less healthy food [31] and muted colours on packaging—as well as the colours green or brown—with healthy choices [24,31].

Few studies examine the persuasive power of branding on packaged foods and children, although existing evidence suggests that branding may be an effective strategy for influencing taste preferences in young children (aged 2–6). However, in relation to food behaviours (such as food intake), and attitudes around healthfulness, results are mixed and research is limited.

Table 3 outlines the total number of studies included in this scoping review that measured the prevalence or effectiveness of single packaging techniques (content analyses, *n* = 21; other studies, *n* = 23), a number of studies also found mixed results, or that specific techniques were ineffective [14,40,41,43,44,47,50,55,56].

## 4. Discussion

When it comes to food marketing to children, food packaging matters. Cartoon characters, premium offers, appeals to “fun”, bright colours, unusual product names or flavours, and direct references to “kid” in the product or brand name all represent persuasive techniques for cueing children and parents to the fact that a packaged food is specifically for kids. The proliferation of “fun” kids food throughout the supermarket has been observed by several studies, with concerns raised over the impact of these marketing strategies on children’s dietary habits, health, relationships with food, and negotiations with commercial culture [23,27,30,66]. Food packaging has been shown to influence young children’s taste preferences [32,62,64] and product preferences [42,49,56], just as has been found in studies tracking the impact of food marketing (more broadly defined) on children’s diets [7,67].

### 4.1. Considering Nutritional Quality and Nutrient Profiling Criteria

Not surprisingly, it is the nutritional quality of these foods that drives much of the discussion when it comes to child-oriented food packaging. Early interest in food packaging and children may have commenced in the marketing literature, with scholars in the late 1970s seeking to find the best route to promote cereals to kids, but when the public health community turns its attention to the food promotion directed at children in the supermarkets some 28 years later [18], it brings a radically different focus. Questions related to the nutritional quality of packaged foods dominate, with studies classifying 97% of child-directed supermarket products as ultra-processed [36] and 89% as “unhealthy” [9,15,22,23,30]. Certain nuances across studies are worth mentioning, however. First, is the issue of the types of foods selected for analysis. Chacon et al. (2013) reported that 97% of the 106 child-oriented snack foods analysed from convenience stores in Mexico were “less healthy” [17]. However, these researchers identified 826 child-oriented snack food, and it is unclear why only 12% of the sample was assessed for nutritional quality, or how the 106 products were selected out of the 826 identified. One equally might assume that convenience stores stock less healthy products than grocery stores. More telling are the content analyses that assess all child-targeted packaged products. That studies providing a comprehensive review of such products report that 89% are unhealthy [9,15,22,23,30] must give us pause for thought. 

When assessing nutritional quality, attention must also be placed on the nutrient profiling model used. Nutritional standards evolve over time, but different models can give a radically different picture of the healthfulness of the food products assessed. For example, when the exact same sample of 374 child-directed supermarket products was analysed using three different nutrient profiling models, the percentage of products permitted to be marketed to children varied dramatically, from 3% of products permitted to 29% [68]. A subsequent study, applying five different nutrient criteria to the same child-directed products, reported similar results [69]. Studies such as these underscore the critical nature of the nutrient criteria used, given the remarkable span between the number of products permitted to be marketed to children depending on the nutrient profiling model—from one in 50 products permitted to one in four. The number of products classified as unhealthy changes depending on the criteria used. The visible package, however, remains the same.

### 4.2. Considering the Power of Child-Targeted Packaged Foods: Importance and Nuance regarding Marketing Techniques 

The “power” of child-oriented packaging is a culmination of various techniques used to persuade, from cartoon characters and incentives to bright colours and product shape. Yet the picture we have of the actual power of these various techniques remains incomplete because the studies track different techniques. At issue here is *persuasive* versus *pervasive* when it comes to techniques. Across all studies, cartoon characters and other endorsers are the most commonly tracked. Since they are most tracked the impression is that they are the most persuasive with children. However, is this in fact the case? Of the 13 studies that examine the impact of cartoon characters on children’s attitudes and behaviors, nine of them report that cartoons on food packages positively influence children’s desire for and “liking of” the product. However, five of these nine studies capture younger age ranges (ages 4-5 [43]; 3-6 [61]; 4-8 [49]; six [65]; and 4-10 [with an average participant age of six]) [44, study #1]. This age range becomes important when viewed in light of the studies that did not find that cartoon characters on packaging impacted children. 

For instance, Ogle and colleagues [56] examined the influence of cartoon media characters on children’s attention to and preference for food and beverage products. Using a computer game, 149 children aged 6–9 viewed 60 pairs of similar products. The same products were displayed both with and without licensed characters on the packaging, while an eye tracking camera recorded how much time children gazed at each package. Children were asked to choose which product they preferred from each pair. While the researchers found that children paid more attention to packages containing licensed characters—that is, their gaze rested longer on packages with characters—they did not *prefer* those packages. Contrary to expectations, Ogle et al. report, “children chose products without characters approximately 62% of the time” [56] (p. 266). The character “reduced the likelihood of a child choosing that product relative to the same product without a character” [56] (p. 268). More significantly, choices differed according to age, sex and the particular licensed character viewed. Older boys and girls, those aged 8-9, were “less likely to choose products with characters on them than without”—a negative finding that was “especially pronounced” when the character was Dora the Explorer [56] (p. 268). In Ogle’s study, only the younger boys (aged 6-7) were “more likely to prefer a product of the same healthfulness with a character”—and only when the character was SpongeBob SquarePants or Lightning McQueen (and not Dora the Explorer) [56] (p. 268).

We dwell on Ogle et al.’s study because it has implications for the other studies that track the impact on children of cartoon characters on packaged foods. As mentioned, over half of the studies that reported that cartoons had a positive impact studied only younger children—the very ages Ogle et al. found “more likely to prefer a product of the same healthfulness” with a specific character [56] (p. 268). If these studies had also tracked older children, would they have reported a different result when it comes to the impact of cartoons? Perhaps. Consider an experimental study that tracked responses by age: it found that when evaluating labels of sponge cake, cartoon characters were more important than nutrient claims to children aged 6-9. However, the exact opposite was found for children aged 10–12 [10]. When these same children evaluated labels of yogurt, moreover, all of them identified the nutrient claim as more important than the cartoon when selecting their “preferred label” [10] (p. 5). Similarly, qualitative research with children has revealed that younger children (age 6-7) prefer food packaging with licensed cartoon characters over those without, but older children (ages 8-12) rejected packages that they felt were “too kiddie” for them [24]. 

Alongside considerations regarding both cartoons (as the most pervasive technique tracked, not necessarily the most persuasive) and age, other noteworthy issues emerge from the literature. First is the issue of defining terms. Cartoon character, as defined in the literature, may capture everything from generic cartoon imagery and brand mascots to cartoons licensed from entertainment media companies. This means we do not have a clear picture of the relative power of each of these types—and they should not be assumed to be synonymous. The relative “power” of Scooby Doo, for instance, may differ from that of a generic cartoon bird for children. Impactful differences also may exist between similar types of cartoons. For example, the handful of studies that specifically look for such differences reveal that not all licensed characters are equally persuasive, and their “power” as a technique changes according to age, gender and the specific character used [24,56]. Focus groups with children reveal that children associate licensed characters on packages (such as Dora the Explorer and Elmo) with unhealthy food—and that this association is more pronounced with older children [31]. Recent experiments with children and familiar licensed characters (LC) add to the complexity, reporting mixed results with respect to their influence [44]: namely, that children are “more likely to choose food in a package with a LC than the same type of food without” [44] (p.226), but will select a “more indulgent” food over a healthy one, irrespective of the presence of a LC. Beyond this, the presence of a LC on a package does not affect consumption. In light of these findings, more attention to the specific types of cartoons tracked would be useful. 

Similar considerations need to be made with respect to other techniques that contribute to the power of child-targeted food packaging. For instance, while packaging imagery and colour were found to influence children’s perceptions and food preferences, the handful of studies that spoke to this in fact assessed examine a range of different visual and colourful elements. Consequently, it is difficult to compare across results. Of these studies, moreover, the visual elements were frequently assessed in tandem—for example, suggesting that imagery and colour impacts children’s choices—which means that it remains unclear which element is more relevant. In several instances, colour is simply discussed in a general sense, as “colour” or “bright colours”. Yet, the precise colours preferred by children (with the exception of colour associations with healthy food, such as green) are not tracked.

A few final points on “power” are worth noting. While the content analyses can work to provide a snapshot of the range and frequency of techniques used, greater consistency across research studies in terms of the specific techniques captured would be useful. Documenting the size of the main persuasive technique used on the package, as one study did [17], would provide valuable insight into what the food industry considers most salient when it comes to targeting children. Tracking the use of marketing techniques over time (as did two studies [30,38]) would reveal changes in the landscape of packaged foods designed to appeal to children. Finally, exploring the impact of more types of techniques (such as incentives, colour, etc.), and nuance between those techniques (i.e., types of cartoon characters) on different ages would provide rich fodder for understanding the true power of child-directed food packaging. 

### 4.3. Strengths and Limitations

This is the first systematic scoping review to assess the published literature on food packaging targeted at children, and to assess that literature for nutritional quality, types of foods examined, impact, and persuasive appeals. Strengths of this article include its comprehensive nature (including all published research and grey literature) and its innovation in capturing the treatment of persuasive power when it comes to child-targeted food packaging. As our goal was to explore the scope of literature on this topic (in order to identify trends and research gaps), we did not evaluate the quality of their evidence. 

## 5. Conclusions

Food packages are portable advertisements, communicating powerful messages to children. The majority of child-directed packaged foods are of poor nutritional quality, suggesting that the very foods designed to appeal to kids could work to compromise their long term health. Food packaging employs powerful techniques to attract children and more attention on these persuasive techniques, and their specific impact on children, is warranted. While much attention has been placed on the marketing of food to children more broadly, a careful examination of the literature on child-directed packaging provides new food for thought—and for policy. 

## Figures and Tables

**Table 1:** Summary of main characteristics of included studies (*n* = 57).

Methods	Object of Study	Child Population Age Ranges	Outcomes Measured *
Content Analysis (22) Experimental (20) Mixed Methods (5) Focus Groups (4) Surveys (2) Interviews (1) Observational (1) Critical Research Paper (2)	Physical Food Packaging (42) Photo images of food packaging (hard copy) (8) Digital images of food packaging (5) Not applicable (2)	Between 2-12 years (24) No age range provided (14) Under and/or over 2-12 years (9) Children & parents (7) Children, teenagers & adults (3)	Exposure (25) Attitudes (22) Behaviours (11) Cognitive (3) Physiological (1) Not applicable (2)

* some studies measure more than one outcome.

**Table 2:** Content analyses that use nutrient profiling criteria (*n* = 21) and key findings.

Study	Criteria Used	Product Sample Size	Food Types Analyzed	Key Findings on Nutritional Profile of Sample	Most Prevalent Food Type in Sample
Chapman et al., 2006 [18]	New South Wales (NSW) Healthy School Canteen Strategy (NSW Dept. of Health and NSW Dept. of Education and Training) & CHOICE magazine (Australian Consumers Association)	231	Sweet biscuits, snack foods, confectionery, chips/savoury snacks, cereals, dairy snacks, ice cream	82% of promotions used to sell unhealthy foods	Confectionery (35% of sample)
Elliott, 2008 [22]	Center for Science in the Public Interest (CSPI) criteria for ‘Poor Nutritional Quality’ (PNQ)	367	Dry goods (i.e., cereals, fruit snacks, drinks), meat, dairy, refrigerated and frozen, beverages, produce	89% of products are poor nutritional quality	Dry goods (61% of sample)
Elliott, 2008 [23]	Center for Science in the Public Interest (CSPI) criteria for ‘Poor Nutritional Quality’ (PNQ)	367	Dry goods, dairy, produce, refrigerated and frozen foods	89% of products are poor nutritional quality	Dry goods (61% of sample)
Page et al., 2008 [57]	Nutrition info (grams of sugar per serving, calories per serving, percent calories from sugar)	122	Ready-to-eat cereals	Mean % of calories from sugar: 34%	Not applicable (entire sample is cereal only)
Harris et al., 2010 [38]	Institute of Medicine (IOM) Nutrition Standards for Foods in Schools guidelines (US)	397	Cereal, fruit snack, meal products, frozen desserts, candy, cookies, other breakfast products, yoghurt and yoghurt drinks, crackers, juice and juice drinks, savoury snacks, fruit and vegetables, other	18.4% of products classified as healthy	Cereal (19% of sample)
Hebden et al., 2011 [39]	Food Standards Australia and New Zealand (FSANZ) Nutrient Profiling Scoring Criterion	352	Chocolate, confectionery, high sugar/high fat spreads, yogurt, cheese, milk, snack food, breakfast cereals, noodles, ice cream, iced confection, sweet and savoury baked goods, sugar-sweetened beverages, powdered flavour additives, canned and frozen meals, fruit and vegetables products, meat/poultry/fish/eggs	74% of products were less healthy	Chocolate & confectionary (36% of sample)
Bragg et al., 2012 [15]	Nutrient Profiling Model (UK)	102	Beverages, snacks, cereal, dessert, condiments, bread, dairy, meat	88.7% of food products were unhealthy	Beverages (48% of sample)
Elliott, 2012 [26]	Center for Science in the Public Interest (CSPI) criteria for ‘Poor Nutritional Quality’ (PNQ) *	354	Dry goods, dairy, produce, frozen food	91% of “regular” child-oriented products have high levels of sugar, fat or sodium 65% of “better-for-you” child oriented products have high sugar, fat or sodium	Dry goods (63% of sample)
Elliott, 2012 [27]	American Heart Association recommendations on sugar	354	Dry goods, dairy, produce, frozen/refrigerated foods	73% of foods derive over 20% of their calories from sugar	Dry goods (67% of sample)
Mehta et al., 2012 [53]	Australian Guide to Healthy Eating	157	Core products (canned/packaged meals, meat and meat alternatives, fruits and fruit products, vegetables and vegetable products, breads/cereal/rice/pasta/noodles, dairy, water), Non-core products (chocolate and confectionery, cakes/muffins/biscuits/pies/pastries, snack foods, fast-food restaurant meals, soft drinks, ice cream and iced confection, sugary breakfast cereals, fruit juice and fruit drinks, frozen/fried potato products, baby and toddler food)	75% of products are non-core foods	Confectionery and chocolate (27% of sample)
Chacon et al., 2013 [17]	Nutrient Profiling Model (UK)	69 (Note: Study identified 106 products, but only 69 were analyzed.)	Savory snacks, pastries and cookies, sweetened beverages (i.e., fruit drinks, energy drinks, sports drinks), soft drinks, dairy products, cereals, ice cream and frozen desserts, light soft drinks, fruit and vegetable snacks or water.	97.1% of food products were unhealthy	Pastries & cookies (37.5% of sample)
Devi et al., 2014 [20]	Food Standards Australia and New Zealand (FSANZ) Nutrient Profiling Scoring Criterion	247	Breakfast cereals (Biscuits and bites, brans, bubbles, flakes and puffs, cereal for kids, muesli, oats)	Kid’s cereal had lower serving size, higher sugar and energy content compared to other cereals	Not applicable (entire sample is cereal only)
Giménez et al., 2017 [36]	Pan American Health Organization Nutrient Profile Model	180	Candy and chocolate, cookies and pastries, dairy products, breakfast cereals, instant foods, soft drinks and juices, savory snacks, frozen ready-to-eat foods, other (meat product, fruit puree, mayonnaise)	97% of products are ultra-processed	Candy and chocolate (25% of sample)
Mediano Stolze et al., 2018 [51]	Nutrition info (total sugars and energy recorded, tax status also recorded–Chilean law: 18% tax if >6.25 g of sugar, 10% tax if < 6.25 g of sugar)	1005	Beverages (plain waters, sports drinks and flavoured waters, soft drinks with sugar, soft drinks without sugar, 100% fruit juice, fruit-flavoured drinks, powder drinks, plains milks, milk-based beverages)	42% of beverages fall into 10% tax rate (moderate sugar, less than or equal to 6.25 mg/100 mL); 27% of beverages fall into 18% tax rate (more than 6.25 mg/100 mL); 31% are not taxed.	Fruit-flavoured drinks (29% of sample)
Pulker et al., 2018 [60]	NOVA System	230	Breakfast cereals, snacks and confectionery items, selected beverages, condiments, liquid breakfast meal replacements	94% of products are ultra-processed	Not provided
Soo et al., 2018 [63]	Nutrient Profiling Model (UK)	106	Breakfast cereals	NPI mean score for cereals is 10.5 (less healthy)	Not applicable (sample is cereal only)
Aerts & Smits, 2019 [9]	Nutrient Profiling Model (UK)	372	Savoury spreads, dairy products, chocolate, cacao powder, cereals, soft candy, cookies, cereal bars, sweet spreads, pasta, ice cream, potato products, fish sticks, crisps, hard candy	89.2% of food products were unhealthy	Candy (18% of sample)
Chen et al., 2019 [19]	WHO recommendations on sugar, saturated fatty acid (Note: This study also uses the Taiwan Ministry of Health and Welfare recommendations on sugar, fat, saturated fatty acids.)	607	Snacks (cookies, breads, RTE cereals, puddings or jellies), Drinks (fruit/vegetable drinks, flavoured milks, fermented milks, soy and rice milks, milk teas).	80% of snacks high in sugar, 54% high in fat 98% of drinks high in sugar, 39% high in saturated fatty acids	Cookies (29% of sample)
Elliott, 2019 [30]	Center for Science in the Public Interest (CSPI) criteria for ‘Poor Nutritional Quality’ (PNQ) & WHO REGIONAL OFFICE FOR EUROPE model	354/374	Dairy, dry goods, produce, meat, refrigerated and frozen foods, beverages	88.7% of products are poorly nutritious in 2009 86.9% of products are poorly nutritious in 2017 88% of products would not be permitted to be marketed to children (in 2009 and 2017)	Dry goods (64% of sample)
García et al., 2019 [35]	OFCOM Nutrient Profiling Model	332	Ready-to-eat cereals, cereal bars, fruit juices, juice drinks, smoothies, dairy and dairy alternatives, ready meals, fruit snacks	41% of products are less healthy	Dairy and dairy alternatives (23% of sample)
Mediano Stolze et al., 2019 [52]	Nutrition info (sugars, fats, sodium, energy–“high-in”–exceeds 2016 Chilean nutrient thresholds–vs. “non high-in”)	168/146	Spanish Language Breakfast Cereals (Ready-to-eat cereals: flakes, puffs, muesli, granola, fibre cereals; Not-RTE cereals: traditional and instant oats)	79% of products pre-implementation are high-in 59% of products post-implementation are high-in	Not provided

* This study also uses the American Heart Association recommendations.

**Table 3:** Summary of findings comparing most prevalent techniques with most effective techniques.

Most Prevalent Packaging Techniques in Content Analyses (n = 21)	Most Effective Packaging Techniques in All Other Studies (n = 23)
**CARTOON CHARACTERS (generic cartoon imagery, brand mascots, licensed characters) (n = 12)** -Cartoon images (69.3% of sample) [26] -Cartoon images (i.e., anthropomorphized animal or figure, or cartoon child) (86%) [27] -Cartoon imagery (licensed or unlicensed) (92%) [35] -Character strategies (i.e., human youth, fantastical non-youth, superhuman, personified object) (30%, 21% of sample in two different time periods) [52] -Cartoon character (76%) [36] -Endorser (animated character) (59.4%) [9] -Promotional characters (i.e., brand mascots, cartoon characters and animals/creatures) (92.5%) [17] -Cartoon/company owned promotional characters (17%) [20] -Company-owned characters (90%) [39] -Cartoon characters (brand mascots) (48%) [18] -Spokes-characters (brand specific) (31%) [63] -Third-party licensed characters (71% over 3 year time period) [38] **PACKAGE LETTERING (FONT) (n = 3)** -Cartoonish script or crayoned font (84%) [22] -Cartoonish script or crayoned font (84%) [23] -Child-appealing font (86% and 94% in two different time periods) [30] **PACKAGING IMAGERY & COLOUR (n = 2)** -Semiotics (i.e., graphics, cartoons, celebrities, claims about health and nutrition) (99%) [53] -Cute pictures or bright colours (60%) [19] **CROSS-PROMOTION (n = 1)** -Tie-ins for TV and movies (72% and 63.5%, respectively) [37] **EMOTIONAL APPEAL (1)** -Emotion and fun (11%) [51] **INFORMATIONAL APPEAL (n = 1)** -“Direct to product/company website” instruction (99.2%) [57] **THEME (n = 1)** -Sports References (i.e., image or text relating to sports organizations, athletes, teams, depictions of physical activity, sports equipment/environments) (34% of products with sports references are also child-targeted) [15] Note: One study does not offer data on most prevalent single technique.	**CARTOON CHARACTERS (cartoon imagery, brand mascots/trade characters, licensed characters) (n = 9)** -impacts food intake for children ages 4–5 [43, study #3] -influences food choice for children aged 6 [65]; and children ages 4–10 [44, study #1] -influences food preference for children ages 6–9 [10] -impacts taste preference for children ages 8–10 [33] -influences taste preference and food choice for children ages 4–8 [49] -influences taste preference and food choice in children ages 3–6 [61] -influences taste preference and food preference for children ages 4–6, and food preference for children 4–6 and 7–9 [45] -influences liking scores for children ages 9–13 [11] **PACKAGING IMAGERY & COLOUR (n = 8)** -influences perceptions of fun and purchase intent for children ages 10–14 [59] -influences food preference for children ages 7–9 [13] -influences food preference ad perceptions of healthfulness for children ages 9–13 [42] -influences perceptions of healthfulness in children in grades 1–6 [16,24,31]; children ages 7–12 [29] -influences favourite packaging and perceptions of healthfulness for children ages 7–8, 9–10, 11–13 [46] **BRANDING (i.e., brand names, logos) (n = 6)** -impacts food intake in overweight children age 4–6 [34]; overweight children ages 4–6 [43, study #1] -influences taste preference for children ages 3–5 [61]; children age 3–6 [64]; children ages 2–5 [32] -influences perceptions of healthfulness in children ages 6–7, 7–11 [48] **INCENTIVES (i.e., premiums) (n = 1)** -influence food choice for children ages 3–12 [12] **OTHER ENDORSERS (i.e., sports celebrities) (n = 1)** -impacts food choice for boys grades 5 & 6, mean age 11 years [21] Note: Excludes critical research papers that do not offer findings on technique effectiveness (n = 2), studies with mixed results or that found techniques to be ineffective (n = 9), and mixed methods studies measuring exposure (n = 2).

## References

[1] Clark H., Coll-Seck A.M., Banerjee A., Peterson S., Dalglish S.L., Ameratunga S., Balabanova D., Bhan M.K., Bhutta Z.A., Borrazzo J. (2020). A future for the world’s children? A WHO-UNICEF-Lancet Commission. Lancet.

[2] World Health Organization (2010). Set of Recommendations on the Marketing of Foods and Non-Alcoholic Beverages to Children. World Health Organization: Geneva. http://apps.who.int/iris/bitstream/handle/10665/44416/9789241500210_eng.pdf;jsessionid=4DA284276CF8B522B7F886DBDF6776D8?sequence=1.

[3] Taillie L.S., Busey E., Stoltze F.M., Carpentier F.R.D. (2019). Governmental policies to reduce unhealthy food marketing to children. Nutr. Rev..

[4] Hastings G., Stead M., McDermott L., Forsyth A., MacKintosh A.M., Rayner M., Godfrey C., Caraher M., Angus K. (2003). Review of research on the effects of food promotion to children. Glasgow: University of Strathclyde, Centre for Social Marketing. http://www.food.gov.uk/news/newsarchive/2003/sep/promote.

[5] Elliott C., Truman E. (2019). Measuring the power of food marketing to children: A review of recent literature. Curr. Nutr. Rep..

[6] World Health Organization (2012). A framework for implementing the set of recommendations on the marketing of foods and non-alcoholic beverages to children. Geneva: World Health Organization. https://apps.who.int/iris/bitstream/handle/10665/80148/9789241503242_eng.pdf?sequence=1.

[7] Smith R., Kelly B., Yeatman H., Boyland E. (2019). Food marketing influences children’s attitudes, preferences and consumption: A systematic critical review. Nutrients.

[8] Haddaway N.R., Collins A.M., Coughlin D., Kirk S. The role of Google Scholar in evidence reviews and its applicability to grey literature searching. PLoS ONE.

[9] Aerts G., Smits T. (2019). Child-targeted on-pack communications in Belgian supermarkets: Associations with nutritional value and type of brand. Health Promot Int..

[10] Ares G., Arrúa A., Antúnez L., Vidal L., Machín L., Martínez J., Giménez A. (2016). Influence of label design on children’s perception of two snack foods: Comparison of rating and choice-based conjoint analysis. Food Qual. Prefer..

[11] Arrúa A., Vidal L., Antúnez L., Machín L., Martínez J., Curutchet M.R., Ares G. (2017). Influence of label design on children’s perception of 2 snack foods. J. Nutr. Educ. Behav..

[12] Atkin C.K. (1978). Observation of parent-child interaction in supermarket decision-making. J. Mark..

[13] Atighi Lorestani E., Khalili M. (2019). Using gamified packaging to increase child influence on family consumption of healthy foods. J. Package Technol. Res..

[14] Bezaz N. (2014). The impact of packaging colour on children’s brand name memorization (7-12 years old). Int. J. Retail. Distrib. Manag..

[15] Bragg M.A., Liu P.J., Roberto C.A., Sarda V., Harris J.L., Brownell K.D. (2013). The use of sports references in marketing of food and beverage products in supermarkets. Public Health Nutr..

[16] Brierley M., Elliott C. (2017). Transparent choices: Communicating packaged food content to children. Vis. Commun..

[17] Chacon V., Letona P., Barnoya J. (2013). Child-oriented marketing techniques in snack food packages in Guatemala. BMC Public Health.

[18] Chapman K., Nicholas P., Banovic D., Supramaniam R. (2006). The extent and nature of food promotion directed to children in Australian supermarkets. Health Promot Int..

[19] Chen M.C., Chien Y.W., Yang H.T., Chen Y.C. (2019). Marketing strategy, serving size, and nutrition information of popular children’s food packages in Taiwan. Nutrients.

[20] Devi A., Eyles H., Rayner M., Ni Mhurchu C., Swinburn B., Lonsdale-Cooper E., Vandevijvere S. (2014). Nutritional quality, labelling and promotion of breakfast cereals on the New Zealand market. Appetite.

[21] Dixon H., Scully M., Niven P., Chapman K., donovan R., Martin J., Baur L.A., Crawford D., Wakefield M. (2014). Effects of nutrient content claims, sports celebrity endorsements and premium offers on pre-adolescent children’s food preferences: Experimental research. Pediatr. Obes..

[22] Elliott C. (2008). Assessing ‘fun foods’: Nutritional content and analysis of supermarket foods targeted at children. Obes. Rev..

[23] Elliott C. (2008). Marketing fun foods: A profile and analysis of supermarket food messages targeted at children. Can. Public Policy.

[24] Elliott C. (2009). Healthy food looks serious: How children interpret packaged food products. Can. J. Commun..

[25] Elliott C. (2010). Eatertainment and the (re)classification of children’s foods. Food Cult. Soc..

[26] Elliott C. (2012). Packaging health: Examining “better-for-you” foods targeted at children. Can. Public Policy.

[27] Elliott C. (2012). Packaging fun: Analyzing supermarket food messages targeted at children. Can. J. Commun..

[28] Elliott C. (2015). ‘Big Food’ and ‘gamified’ products: Promotion, packaging, and the promise of fun. Crit. Public Health.

[29] Elliott C. (2018). Milk in a glass, milk in a carton: The influence of packaging on children’s perceptions of the healthfulness of milk. Int. J. Health Promot. Educ..

[30] Elliott C. (2019). Tracking kids’ food: Comparing the nutritional value and marketing appeals of child-targeted supermarket products over time. Nutrients.

[31] Elliott C., Brierley M. (2012). Healthy Choice?: Exploring how children evaluate the healthfulness of packaged foods. Can. J. Public Health.

[32] Elliott C., Den Hoed R.C., Conlon M.J. (2013). Food branding and young children’s taste preferences: A reassessment. Can. J. Public Health.

[33] Enax L., Weber B., Ahlers M., Kaiser U., Diethelm K., Holtkamp D., Kersting M. (2015). Food packaging cues influence taste perception and increase effort provision for a recommended snack product in children. Front Psychol. Behav. Sci..

[34] Forman J., Halford J.C.G., Summe H., MacDougall M., Keller K.L. (2009). Food branding influences ad libitum intake differently in children depending on weight status. Results of a pilot study. Appetite.

[35] García A.L., Morillo-Santander G., Parrett A., Mutoro A.N. (2019). Confused health and nutrition claims in food marketing to children could adversely affect food choice and increase risk of obesity. Arch. Dis. Child..

[36] Giménez A., Saldamando L., Curutchet M.R., Ares G. (2017). Package design and nutritional profile of foods targeted at children in supermarkets in Montevideo, Uruguay. Cad Saúde Pública..

[37] Grigsby-Toussaint D.S., Moise I.K., Gieger S.D. (2011). Observations of marketing on food packaging targeted to youth in retail food stores. Obesity.

[38] Harris J.L., Schwartz M.B., Brownell K.D. (2010). Marketing foods to children and adolescents: Licensed characters and other promotions on packaged foods in the supermarket. Public Health Nutr..

[39] Hebden L., King L., Kelly B., Chapman K., Innes-Hughes C. (2011). A menagerie of promotional characters: Promoting food to children through food packaging. J. Nutr. Educ. Behav..

[40] Heller R., Martin-Biggers J., Berhaupt-Glickstein A., Byrd-Bredbenner C. (2015). Fruit-related terms and images on food packages and advertisements affect children’s perceptions of foods’ fruit content. Public Health Nutr..

[41] Hota M., Charry K. (2014). The impact of visual and child-oriented packaging elements versus information on children’s purchase influence across various age groups. Int J Retail Distrib Manag..

[42] Kang S.R., Ladjahasan N., Satterfield D., Muratovski G., Vogel C. (2019). Snack food package design: Exploratory study on children’s snack choices and design elements. Design and Living Well.

[43] Keller K.L., Kuilema L.G., Lee N., Yoon J., Mascaro B., Combes A.L., Deutsch B., Sorte K., Halford J.C. (2012). The impact of food branding on children’s eating behaviour and obesity. Physiol. Behav..

[44] Leonard B., Campbell M.C., Manning K.C. (2019). Kids, caregivers, and cartoons: The impact of licensed characters on food choices and consumption. J Public Policy Mark.

[45] Letona P., Chacon V., Roberto C., Barnoya J. (2014). Effects of licensed characters on children’s taste and snack preferences in Guatemala, a low/middle income country. Int. J. Obes..

[46] Letona P., Chacon V., Roberto C., Barnoya J. (2014). A qualitative study of children’s snack food packaging perceptions and preferences. BMC Public Health.

[47] Levin A.M., Levin I.P. (2010). Packaging of healthy and unhealthy food products for children and parents: The relative influence of licensed characters and brand names. J. Consum. Behav..

[48] Maher J.K. (2012). It’s called fruit juice so it’s good for me right?: An exploratory study of children’s fruit content inferences made from food brand names and packaging. J. Bus Res..

[49] McGale L.S., Halford J.C.G., Harrold J.A., Boyland E.J. (2016). The influence of brand equity characters on children’s food preferences and choices. J. Pediatr..

[50] McNeal J.U., Ji M.F. (2003). Children’s visual memory of packaging. J. Int. Consum. Mark..

[51] Mediano Stoltze F., Barker J.O., Kanter R., Corvalán C., Reyes M., Taillie L.S., Carpentier F.R.D. (2018). Prevalence of child-directed and general audience marketing strategies on the front of beverage packaging: The case of Chile. Public Health Nutr..

[52] Mediano Stoltze F., Reyes M., Smith T.L., Correa T., Corvalán C., Carpentier F.R.D. (2019). Prevalence of child-directed marketing on breakfast cereal packages before and after Chile’s Food Marketing Law: A pre- and post-quantitative content analysis. Int. J. Environ. Res. Public Health.

[53] Mehta K., Phillips C., Ward P., Coveney J., Handsley E., Carter P. (2012). Marketing foods to children through product packaging: Prolific, unhealthy and misleading. Public Health Nutr..

[54] Musicus A., Tal A., Wansink B. (2015). Eyes in the aisles: Why is Cap’n Crunch looking down at my child?. Environ. Behav..

[55] Nelson M.R., Duff B.R.L., Ahn R. (2015). Visual perceptions of snack packages among preschool children. Young Consum..

[56] Ogle A.D., Graham D.J., Lucas-Thompson R.G., Roberto C.A. (2017). Influence of cartoon media characters on children’s attention to and preference for food and beverage products. J. Acad. Nutr. Diet..

[57] Page R., Montgomery K., Ponder A., Richard A. (2008). Targeting children in the cereal aisle: Promotional techniques and content features on ready-to-eat cereal product packaging. Am. J. Health Educ..

[58] Pavleen S. (2013). Promoting foods to Indian children through product packaging. J. Compet..

[59] Pires C., Agante L. (2011). Encouraging children to eat more healthily: The influence of packaging. J. Consum. Behav..

[60] Pulker C.E., Scott J.A., Pollard C.M. (2018). Ultra-processed family foods in Australia: Nutrition claims, health claims and marketing techniques. Public Health Nutr..

[61] Roberto C.A., Baik J., Harris J.L., Brownell K.D. (2010). Influence of licensed characters on children’s taste and snack preferences. J. Pediatr..

[62] Robinson T.N., Borzekowski D.L.G., Matheson D.M., Kraemer H.C. (2007). Effects of fast food branding on young children’s taste preferences. Arch. Pediatr. Adolesc. Med..

[63] Soo J., Letona P., Chacon V., Barnoya J., Roberto C.A. (2016). Nutritional quality and child-oriented marketing of breakfast cereals in Guatemala. Int. J. Obes..

[64] Tim L.S., Beevi Z., Yeap R. (2014). Effects of fast-food branding on children’s taste preferences. Southeast Asia Psych J..

[65] Ülger B. (2009). Packages with cartoon trade characters versus advertising: An empirical examination of preschoolers’ food preferences. J. Food Prod. Mark..

[66] Elliott C. (2018). Beauty and the Banana: It’s a commercial promotion, not a public health campaign. Can. J. Public Health.

[67] Boyland E., Whalen R., Christiansen P., Mcgale L., Duckworth J., Halford J., Clark M., Rosenberg G., Vohra J. (2018). See It, Want It, Buy It, Eat It: How Food Advertising is Associated with Unhealthy Eating Behaviours in 7–11 Year Old Children.

[68] Elliott C., Scime N.V. (2019). Nutrient profiling and child-targeted supermarket foods: Assessing a “made in Canada” policy approach. Int. J. Environ. Res. Public Health.

[69] Mulligan C., Christoforou A.K., Vergeer L., Bernstein J.T., L’Abbé M.R. (2020). Evaluating the Canadian Packaged Food Supply Using Health Canada’s Proposed Nutrient Criteria for Restricting Food and Beverage Marketing to Children. Int. J. Environ. Res. Public Health.

